# Infectiousness of Tuberculosis

**Published:** 1893-01

**Authors:** 


### INFECTIOUSNESS OF TUBERCULOSIS.

According to the latest advices from Germany, where Dr. Koch's investigations in regard to tuberculosis have given a special impetus to the study of the disease, the danger arising from direct contact with consumptive patients has been somewhat overestimated, notwithstanding the infectious nature of the malady.

More recent investigation has shown that the bacilli which are coughed up in the sputa of consumptives, and which are hence present in greater or less numbers in the mucus of the mouth and nose, are for the most part lifeless and inactive.

This demonstration, while it will bring comfort to many people thus afflicted, will probably tend to emphasize the fact of a family predisposition to the disease; and this in turn will doubtless lead to a fuller study of the peculiar physical and anatomical characteristics of special families and individuals, who are at present vaguely termed by the profession "subjects with a predisposition to tuberculosis."

The study ought to lead by a natural process to the correction of such anatomical characteristics, or "build of frame," by means of special methods of chest or general exercises, by the correction of habitual errors of breathing, and by other corrective and cautionary measures.

But if the dangerous character of consumptive expectorations has perhaps been overestimated, the danger is none the less real, and the precautions which patients and nurses have been urged to exercise for the public safety should still be continued. Sputa should be burned; spitting in the streets or public places should be avoided, and great care should be taken to prevent collections of dust in any form in the rooms occupied by consumptives. This last precaution is of importance for the patient as well as for his family.—*Youths' Companion.*

